# Perceptual Improvement of Lexical Tones in Infants: Effects of Tone Language Experience

**DOI:** 10.3389/fpsyg.2017.00558

**Published:** 2017-04-11

**Authors:** Feng-Ming Tsao

**Affiliations:** Department of Psychology, National Taiwan UniversityTaipei, Taiwan

**Keywords:** infant lexical tone perception, pitch contour, native and non-native speech perception, developmental trends, Mandarin lexical tones

## Abstract

To learn words in a tonal language, tone-language learners should not only develop better abilities for perceiving consonants and vowels, but also for lexical tones. The divergent trend of enhancing sensitivity to native phonetic contrasts and reduced sensitivity to non-native phonetic contrast is theoretically essential to evaluate effects of listening to an ambient language on speech perception development. The loss of sensitivity in discriminating lexical tones among non-tonal language-learning infants was apparent between 6 and 12 months of age, but only few studies examined trends of differentiating native lexical tones in infancy. The sensitivity in discriminating lexical tones among 6–8 and 10–12 month-old Mandarin-learning infants (*n* = 120) was tested in Experiment 1 using three lexical tone contrasts of Mandarin. Facilitation of linguistic experience was shown in the tonal contrast (Tone 1 vs. 3), but both age groups performed similar in the other two tonal contrasts (Tone 2 vs. 4; Tone 2 vs. 3). In Experiment 2, 6–8 and 10–12 month-old Mandarin-learning infants (*n* = 90) were tested with tonal contrasts that have pitch contours either similar to or inverse from lexical tones in Mandarin, and perceptual improvement was shown only in a tonal contrast with familiar pitch contours (i.e., Tone 1 vs. 3). In Experiment 3, 6–8 and 10–12 month-old English-learning infants (*n* = 40) were tested with Tone 1 vs. 3 contrast of Mandarin and showed an improvement in the perception of non-native lexical tones. This study reveals that tone-language learning infants develop more accurate representations of lexical tones around their first birthday, and the results of both tone and non-tone language-learning infants imply that the rate of development depends on listening experience and the acoustical salience of specific tone contrasts.

## Introduction

Perceptual sensitivity to consonants and vowels undergoes rapid changes during the first year of life. Infants start with a universal capacity to distinguish the phonemes of native and foreign languages (Eimas et al., 1971; Streeter, 1976), and demonstrate improved sensitivity in discriminating native phonemes occur in infants between 6 and 12 months of age (Kuhl et al., 2006; Tsao et al., 2006). Similar to consonants and vowels, lexical tones distinguish lexical meanings of syllables in tonal languages: the most well-known example of a tone language is Mandarin Chinese, which boasts the largest number of first-language speakers worldwide (Lewis et al., 2015). The developmental trends of infants distinguishing consonants and vowels from both native and foreign languages are well-documented (Werker et al., 2012), but only few studies have explored the developmental trajectories of lexical tones in non-tonal language-learning infants (Mattock and Burnham, 2006; Mattock et al., 2008; Yeung et al., 2013; Liu and Kager, 2014; Singh and Fu, 2016; Singh et al., 2016). It remains unclear whether infants learning a tonal language as their first language *improve* in their sensitivity in distinguishing lexical tones during the second half of their first year of life.

There is increasing evidence to suggest that infants acquire detailed information of their native language by listening to and analyzing linguistic inputs during the first year of life (Kuhl et al., 2008; Werker et al., 2012). By 6 months of age, infants engage in a detailed analysis of the distributional properties of the sounds contained in their ambient language, which alters their perception such that they tend to focus more on native-like phonetic processing (Kuhl et al., 1992; Maye et al., 2008). By 10–12 months of age, the developmental change in the phoneme perception of infants is apparent. There is a steep decline in the discrimination of non-native phonemes (Werker and Tees, 1984; Palmer et al., 2012) and an improvement in that of native phonemes (Kuhl et al., 2006; Tsao et al., 2006), reflecting changes that depend on linguistic experience. Although, rapid changes in differentiating consonant contrasts between 6 and 12 months age were reported in numerous studies, few studies have reported the maintenance of perceptual sensitivity. For example, 10–12 month-old English-infants tested on their ability to discriminate the /d/ vs. /ð/ contrast of English performed similarly to 6–8 month-old infants of the same language (Polka et al., 2001). The language-specific pattern of differentiating English /d/ vs. /ð/ contrast emerged later than 12 months of age, when 4-year-old English-speaking children performed better than French-speaking children of the same age in distinguishing the English /d/ vs. /ð/ contrast (Sundara et al., 2006).

On perceptual development of phonetic segments, several theoretical models, such as attunement, perceptual learning and maturation theories, have been proposed to interpret effects of language experience on developmental trajectories of speech perception in infancy (Aslin and Pisoni, 1980). Studies that show the perceptual decline in discrimination of non-native consonants and perceptual improvement in discrimination of native consonants have provided greater support to theories of attunement and perceptual learning than other models. With increasing listening experience to the ambient language, attunement theory assumed that phonologically relevant contrasts would be finely tuned, but phonologically irrelevant contrasts would remain broadly tuned or attenuated. In other words, attunement theory predicts three developmental trajectories of discriminating native and non-native phonetic contrasts: facilitation, maintenance, and loss. Perceptual learning theory assumes that development of speech perception depends on frequency of occurrence and relative acoustical discriminability of specific phonetic contrasts, and rate of development could be slow or fast. Despite that attunement theory gains more support than perceptual learning theory, some *hybrid* of theories best describes the development of specific categories of phonetic discrimination (Aslin and Pisoni, 1980). Would the perceptual development trends predicted by attunement theory, perceptual learning theory, or their combination be evident in tonal perception development?

Despite the extensive literature on infant perception of phonetic segments (e.g., vowels and consonants), the developmental trends of lexical tones in tonal and non-tonal language learners have not been fully explored (Singh and Fu, 2016). Nevertheless, some studies have reported mixed findings regarding whether the *perceptual decline* in the discrimination of lexical tones is universal in non-tonal language-learning infants before their second birthday. Some studies have demonstrated a perceptual decline that occurred among English-learning infants between 4 and 9 months of age when discriminating lexical tones of Thai or Cantonese (Mattock and Burnham, 2006; Mattock et al., 2008; Yeung et al., 2013). Compared with French-learning 6-month-old infants, reduced sensitivity to discriminating lexical tones of Thai has also been reported among 10-month-old infants learning the same language (Cabrera et al., 2015). However, 19-month-old English-learning infants were able to discriminate lexical tone contrasts of Mandarin (Hay et al., 2015, Experiment 3). For Dutch-learning infants, they were able to discriminate Mandarin lexical tone contrasts with *larger pitch* differences between 5 and 18 months of age; however, their sensitivity in distinguishing that same tonal contrast with *smaller pitch* difference was reduced between 9 and 15 months of age, and improved at approximately 18 months of age (Liu and Kager, 2014). These studies raised questions regarding whether the experience of listening to a non-tonal language either reduces or maintains infants’ sensitivity in distinguishing lexical tones after 9 months of age, and results of Liu and Kager (2014) suggested that acoustical discriminability of contrasts impacted the development of tone sensitivity.

Reduced sensitivity to lexical tone contrasts among non-tonal language learners reveals that listening to an ambient language shifts the perceptual organization of lexical tones, and partially supports the attunement theory because a loss in sensitivity to tone is predicted by this model. Assessing tone perception among tonal language learners is not only necessary to reveal the developmental trends of differentiating native tone contrasts, but enhanced sensitivity to native tone contrasts is also theoretically required to evaluate attunement theory of speech perception development. In addition to listening to a tonal language, if development of tone perception depends on relative acoustical discriminability of specific tone contrasts, the perceptual learning model assumes that rate of development is slow for infants to distinguish acoustically similar tone contrasts. In other words, facilitation as well as maintenance of differentiating native tone contrasts across ages are predicted by models of speech perception.

It is therefore important to assess whether the native phonological system facilitates or maintains tonal-language learning infants’ sensitivity to native tonal contrasts while non-tonal language learners change their sensitivity to non-native lexical tones. Such an investigation would help construct a better conceptual framework through which the development of native and non-native tone sensitivity could be explored between 6 and 12 months of age. Mandarin-learning infants and Cantonese-learning infants have been reported to show language-specific listening preferences for their native lexical tones at approximately 5 months of age (Yeung et al., 2013). However, it is still unclear whether exposure to a tonal language would either facilitate or maintain infants’ sensitivity in the discrimination of native tone contrasts around their first birthdays.

**FIGURE 1:**
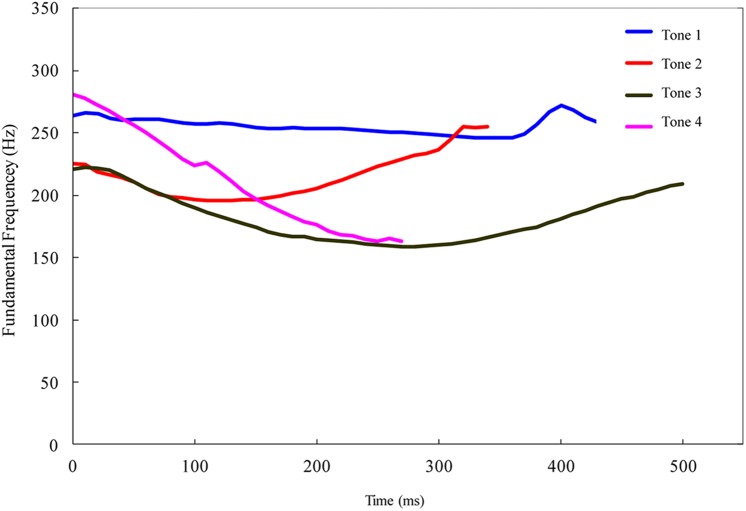
**Pitch contours (fundamental frequency) of lexical tone stimuli in Experiments 1 and 3 to examine tonal perception development in infancy.** Adapted from Tsao (2008).

The rate of tone perception developmental might vary with the relative acoustical salience of tone contrasts. In infant- and child-directed speech, the average heights and contours of the fundamental frequency (F0) distinguish four lexical tones in Mandarin; however, some tones have similar F0 contours (Liu et al., 2009). **Figure 1** illustrates the F0 contours of the four lexical tones in Mandarin. Tone 1 is a high-level tone and Tone 4 is a high-falling tone. The pitch directions of both Tones 1 and 4 are not greatly altered within a syllable. However, Tones 2 (mid-rising tone) and 3 (low-dipping tone) exhibit similar F0 contours in isolated syllables: both have a concave F0 shape. The acoustical similarity between Tones 2 and 3 results in the frequent confusion of this tone contrast by non-tonal language speakers (Wang et al., 1999; So and Best, 2010). In contrast, although Tones 2 and 4 exhibit a similar average F0, they have different F0 contours: a rising F0 contour for Tone 2 and a falling F0 contour for Tone 4. Perceptual discrimination of the Tones 2 and 3 pair is the most difficult for English adult speakers, followed by Tones 2 and 4 pair, and Tones 1 and 3 pair is the easiest (e.g., Wang et al., 1999). For Mandarin-learning children, 3-year-old Mandarin-speaking children easily confuse Tone 3 with Tone 2 compared to other tone pairs (Wong et al., 2005). Acoustical salience of tone contrasts also affects the discrimination of lexical tone in preverbal infants. Tsao (2008) reported that 12-month-old Mandarin-learning infants were more accurate in discriminating the contrast between Tones 1 and 3 than those between Tones 2 and 4 and Tones 2 and 3. Tsao’s (2008) results suggested that the growth rate for distinguishing tone contrasts between 6 and 12 months in Mandarin-learning infants might vary with the acoustical salience of tone contrasts. The acoustical salience of consonant contrasts influences infants’ abilities to differentiate syllable-initial consonants between 6 and 12 months of age (e.g., Narayan et al., 2010). Adopting tone contrasts that vary acoustical salience would be conceptually essential to examine whether the rate of tone perception development depends on both the listening experience with lexical tones and the relative acoustical discriminability of tone contrasts.

Although, both pitch height (measured by the mean fundamental frequency) and pitch direction (measured by the time of pitch direction change or the slope of pitch contour) (Liu et al., 2009; Chandrasekaran et al., 2010) are acoustical correlates of Mandarin lexical tones, the perceptual weights of these acoustical cues vary with speakers’ levels of proficiency in identifying and discriminating lexical tones. For Mandarin speakers, the pitch direction (or pitch contour) is perceptually weighted more heavily than the pitch height. In contrast, English speakers tend to weigh pitch height more than they do pitch direction (Gandour and Harshman, 1978). The perceptual weight difference between the height and direction of pitch also indicates the individual differences among non-tonal language speakers when perceiving lexical tones. English-speaking adults, who are more accurate in labeling the pitch pattern (level, rising, and falling) of lexical tones, also weigh the pitch direction more heavily than they weigh the pitch height (Chandrasekaran et al., 2010). In brief, adult speakers who are able to track pitch contour would exhibit better tone perception of Mandarin tones. In addition to exploring the general trends of differentiating tone contrasts between 6 and 12 months of age, to further examine developmental mechanism of tone perception, it is essential to explore whether infants attune to language-specific pitch contours while improving their perceptual sensitivity to native tonal contrasts.

The acoustical features of lexical tones, i.e., pitch height and contours, are also acoustical parameters of linguistic prosody. Nevertheless, variations of pitch contour *within* syllables do not change the lexical meanings of English syllables; 8- to 12- month-old English-learning infants showed an improvement in their ability to utilize prosodic patterns *between* syllables (i.e., word stress) in the segmentation of words and phrases from continuous speech (Soderstrom et al., 2003; Thiessen et al., 2005; Seidl, 2007). If the improvements in the ability of English-learning infants to process linguistic prosody generalized to pitch features of lexical tones, the accuracy of discriminating lexical tones by English-learning infants might either not decline or even improve for each tonal contrast of a non-native language before their first birthday.

To reiterate, this study aimed to examine developmental trajectories of native and non-native tone perception among infants between 6 and 12 months of age. In addition, this study also explored whether the sensitivity to acoustical features of language-specific lexical tones, such as pitch contours, enhances tone perception around the first birthday. Three experiments were conducted to address these questions. Experiment 1 was designed to explore developmental trends of native lexical tone perception among Mandarin-learning infants. The acoustical salience of lexical tone contrasts refers to the magnitude of the differences between acoustical parameters essential to differentiate lexical tones (i.e., pitch height and contour). The acoustically most salient contrast has the largest acoustical difference, i.e., the Tone 1 vs. 3 contrast. To increase acoustical salience of tonal contrasts, the following tone contrasts were used: Tone 1 vs. Tone 3, Tone 2 vs. Tone 4, and Tone 2 vs. Tone 3. If lexical tone perception underwent a marked change between 6 and 12 months of age, the older Mandarin-learning infants would outperform the younger ones in the discrimination of native lexical tones. However, if rate of development depends on the interaction between listening experience and relative acoustical salience of tone contrasts, developmental trends of differentiating native tone contrasts would vary with tone contrasts. Improved sensitivity to discriminate tone contrasts might be observed for acoustically more salient contrasts, but maintenance of perceptual sensitivity might be shown for acoustically less salient contrasts. Experiment 2 explored whether Mandarin-learning infants relied on language-specific pitch contours to discriminate tonal contrasts, by testing the sensitivity to two tonal contrasts in which whether the tone contrasts were native to Mandarin or not was identified purely by pitch contour. The pitch contours of one tonal contrast were similar to the lexical tones in Mandarin, but contours of the other tonal contrast were inverse of the lexical tones in Mandarin. The assumption of Experiment 2 was that older Mandarin-learning infants would outperform their younger peers in discriminating tone contrasts with pitch contours similar to Mandarin tones. Experiment 3 employed a cross-language design to examine the developmental trends in the perception of non-native lexical tones among 6–8 and 10–12 month-old English-learning infants. The hypothesis was that acoustical salience of tone contrast and improvement of linguistic prosody in English-learning infants around the first birthday would also enhance English-learning infants’ ability in distinguishing tone contrasts with greater acoustical salience. In addition, if the 10–12 month-old Mandarin-learning infants demonstrated higher accuracy in discriminating Mandarin tones than the English-learning infants at the same age, it would indicate that listening to lexical tones provides additional benefits to facilitate the development of lexical tones.

## Experiment 1: Development of Native Lexical Tone Perception

### Method

#### Participants

Two age groups of Mandarin-learning infants (*n* = 120) participated in the study: (a) 10–12-month-olds: Tone 1 vs. 3 (*n* = 20, girls *n* = 10, mean age = 10.96 months, *SD* = 1.23 months), Tone 2 vs. 3 (*n* = 20, girls *n* = 6, mean age = 11.10 months, *SD* = 0.82 months), and Tone 2 vs. 4 (*n* = 20, girls *n* = 9, mean age = 11.12 months, *SD* = 0.74 months); (b) 6–8-month-olds: Tone 1 vs. 3 (*n* = 20, girls *n* = 8, mean age = 7.33 months, *SD* = 0.50 months), Tone 2 vs. 3 (*n* = 20, girls *n* = 8, mean age = 7.32 months, *SD* = 0.44 months), and Tone 2 vs. 4 (*n* = 20, girls *n* = 11, mean age = 7.32 months, *SD* = 0.38 months). Eighteen additional infants failed to complete the testing procedures due to their inability to pass the conditioning. Results of a χ^2^ test on the rates of infants who could not pass the conditioning phase of the tone discrimination procedure indicated neither the age nor tone effect reached significance, at 6–8 months, χ^2^(2) = 0.156, *p* = 0.925, and at 10–12 months, χ^2^(2) = 0.252, *p* = 0.882. The pre-established criteria for inclusion in the study were that infants had no known visual or auditory deficits, were born full term (±14 days from the due date), were delivered without complications, had a normal birth weight (2.5–4.5 kg), and were developing normally. In addition, the members of the infants’ immediate families had no history of hearing loss. Parents were paid NT$ 600 for their child participating in the experiment.

Mandarin-learning infants were recruited either from the lists of names on the House Registry of the Da-An and Chung-Cheng Areas, Taipei City, Taiwan, or through an advertisement notice posted on the Internet. Although Taiwan is a multi-lingual society, Mandarin is the most dominant language spoken in homes. The Mandarin-dominant (or -only) language environment of Taiwanese infants was verified through a language background questionnaire, which was administrated to the caregiver before the study began. This study was carried out in accordance with the recommendations of ‘American Psychological Association ethical standards’ and ‘Research Ethics Committees of National Taiwan University’ with written informed consent from all participants. All parents gave written informed consent in accordance with the Declaration of Helsinki.

#### Stimuli

The speech stimuli were Tone 1 [tɕ^h^i1] (duration = 690 ms), Tone 2 [tɕ^h^i2] (duration = 600 ms), Tone 3 [tɕ^h^i3] (duration = 770 ms), and Tone 4 [tɕ^h^i4] (duration = 482 ms) syllables, recorded in a sound-attenuation booth by a female Mandarin-native speaker with a normal speaking rate, and digitized with the speech analysis software, Computerized Speech Lab (CSL 4400), at a 22050 sampling rate and 16-bit resolution. The use of naturally produced speech stimuli instead of computer synthesized stimuli provided the most natural tokens by which lexical tone sensitivity in infants could be examined. Acoustical salience between tonal contrasts was reported to affect the accuracy of discriminating tonal contrasts among 1-year-old Mandarin-learning infants (Tsao, 2008); this experiment adopted three tone contrasts regarding to the average F0 and F0 contour: (1) the Tone 1 vs. 3 pair was acoustically the most distinct; (2) Tone 2 vs. 3 was acoustically the most similar; and (3) Tone 2 vs. 4 had a moderate acoustical similarity. The duration, average F0, F0 range, and turning point [= (time of the minimal F0 ÷ tone duration) × 100%] are acoustical correlates of lexical tones (Liu et al., 2007). Acoustical correlates of lexical tones were assessed using the speech analysis software Praat (Boersma and Weenink, 2011). For speech stimuli in this experiment, lexical tones were only manifested on vowels. **Figure 1** illustrates the F0 contours of the four lexical tones and **Table 1** lists the acoustical features of lexical tones. The duration of lexical tones is an acoustical correlate of lexical tones in natural speech (Liu et al., 2009) and was preserved in the digitized speech stimuli. The durations of syllable-initial consonant [tɕ^h^] are 238 ms (Tone 1), 240 ms (Tone 2), 216 ms (Tone 3) and 192 ms (Tone 4), respectively. The speech samples were edited with the sound-editing software Sound Forge 7.0 (Sony, 2004) to equalize the root mean square (RMS) levels of each syllable.

**Table 1:** Acoustical parameters of lexical tones in Experiment 1.

Lexical tones	Stimulus duration (ms)	Mean F0 (Hz)	F0 range (Hz)	Turning point (%)
T1	690	256	27	Level
T2	600	215	60	33
T3	770	183	65	50
T4	482	212	121	100

#### Apparatus

Speech stimuli were presented using a personal computer (HP Compaq DC7100). The sounds were amplified (Yamaha RX V350) and delivered to infants in an adjoining sound-treated test room via a loudspeaker (Bowers & Wilkins DM303). Parents and experimenters wore headphones (SONY MDR-CD 280) and listened to music from a CD during the tests, so they could not distinguish between the stimuli presented to the infants. Infants’ responses were monitored in the control room using a digital camera (SONY Handycam PC350) and a video monitor. Operated by an experimenter, who pushed a button on a hand-held switch, the computer used a data acquisition board (National Instrument PCI-6503) to activate the reinforcer and record the infants’ head-turn responses.

#### Test Suite

The test suite consisted of two rooms. In the sound-attenuation test room, an infant was held on his or her parent’s lap, facing forward while an assistant sat at a 90-degree angle to the infant’s right side. An assistant maintained the infant’s attention by manipulating a series of engaging, silent toys to bring the infant’s gaze to midline (straight ahead of the infant). A bank of two visual reinforcers was located at a 90-degree angle to the infant’s left side, and each consisted of a dark Plexiglas box (13″ × 13″ × 13″) containing a commercially available mechanical toy (e.g., a dancing snowman). The toys were not visible until activated, at which point the lights mounted inside the box were illuminated. The visual reinforcers were placed on either side of the loudspeaker, at the infant’s eye level. A camera located in front of the infant fed an image of the test room to the adjoining control room, where an experimenter observed the infant’s behavior.

#### Infant Testing Procedure

The Head-Turn (HT) testing procedure has been previously used to explore developmental changes in consonant perception among infants 6–12 months of age (Kuhl et al., 2006; Tsao et al., 2006). Infants were first trained to produce a head turn for visual reinforcement whenever the “background” speech sound (e.g., [tɕ^h^i1]), which was repeated once every 2 s, would be changed to the “target” speech sound (e.g., [tɕ^h^i3]). Pitch contour of Tones 2 and 3 are acoustically more similar than the other two lexical tones (i.e., Tones 1 and 4), and to reduce the possibility that large acoustical differences between target speech sounds of tonal contrasts would also contribute to the performance differences among tone contrasts, the target tone of each contrast was one of contour tones. Tone 3 was the target tone for the Tone 1 vs. 3 and the Tone 2 vs. 3 contrasts, while Tone 2 was the target tone for the Tone 2 vs. 4 contrast. The experimental protocol required a two-step training phase followed by a Test phase, all of which were computer-controlled. While the speech stimuli were playing in the background, the assistant played with toys to get the infant’s attention and distract the infant’s attention from the speech stimuli.

The first step of the training phase consisted of Conditioning (+ Intensity). During this phase, infants were trained to associate the presentation of the target speech sound with the activation of visual reinforcers. The target sound interrupted the repetitive presentation of the background speech sound, and was presented at a level that was 4 dBA higher than that of the background speech sound. During the training phase, every trial was considered a target trial. The target stimulus was presented three times in a row. The onset-to-onset inter-stimulus interval was 2000 ms. The infant quickly learned to anticipate the visual reinforcer when the speech sound was changed from the background to the target. The infant had to respond to the sound change within 6 s after the first presentation of the target sound in order to watch the visual reinforcement. When the infant correctly anticipated the visual reinforcers with a head turn on two consecutive trials, the test proceeded to the next training phase, Conditioning (- Intensity).

In the Conditioning (- Intensity) phase, the target sound was presented at the same intensity level as the background sound; the infants used only the phonetic difference between the sounds as a cue. All other parameters of the experiment remained the same. The infants needed to correctly produce three anticipatory head turns to advance to the Test phase. Those who failed to pass the two-phase training within 30 trials were excluded from the sample. The speech stimuli were the same in both Conditioned and Test phases, similar to those in other infant studies using the head-turn procedure (Kuhl et al., 2006; Tsao et al., 2006). The Test phase consisted of 30 trials, with an equal number of Change and Control (no-change) trials presented in random order. Infants completed both training and testing phases in about 20 min on the same day.

In all phases of training and testing, trials were initiated by the research assistant, who showed toys to the infants in the test room. The assistant initiated trials when infants appeared ready (focusing on the toys held by the assistant). The experimenter could not hear the stimuli presented during the trials (a computer-controlled gating network cut out the sound during the trial), and was unaware of the type of trial that was automatically selected by the computer. The experimenter judged the head turn and pushed a button on a hand-held switch connected to the computer through the data acquisition board to indicate a head turn. The assistant could not hear the stimuli being presented at any time during the experiment, but was informed that a trial was underway by a small light that was automatically activated for the duration of a trial (out of the infant’s view). This was necessary information for the assistant as she was instructed not to change the toy in the midst of a trial.

### Results and Discussion

**FIGURE 2:**
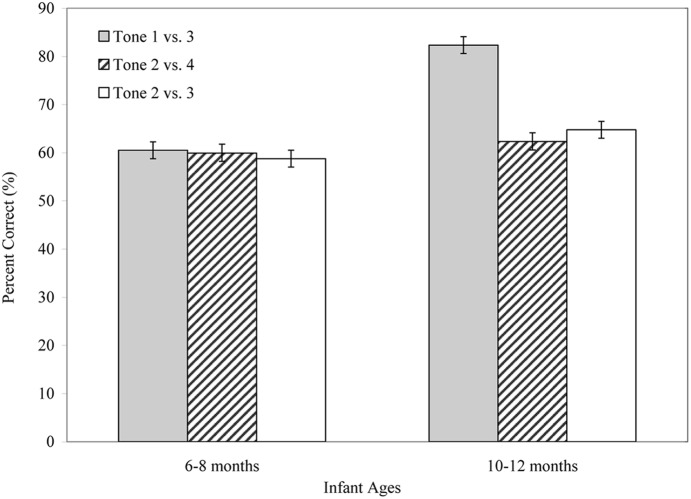
**Mean percentage of the correct responses (+ *SE*) of Mandarin-learning infants in discriminating native lexical tone contrasts at 6–8 and 10–12 months of age**.

An Age × Contrast two-way ANOVA of the percentage of correct responses revealed that 10–12-month-old Mandarin-learning infants (*M* = 69.86%, *SD* = 12.96) performed better than their 6–8-month-old counterparts (*M* = 59.64%, *SD* = 5.74), *F*(1,114) = 51.22, *p* < 0.001, $\eta_{p}^{2}$ = 0.310. Further, perceptual accuracy significantly varied by the tone contrast, *F*(2,114) = 21.55, *p* < 0.001, $\eta_{p}^{2}$ = 0.274. The Age × Contrast interaction was significant, *F*(2,114) = 18.39, *p* < 0.001, $\eta_{p}^{2}$ = 0.244, showing that the developmental trends in the discrimination of lexical tones varied by tonal contrasts. **Figure 2** show the percentage of correct responses by infants aged 6–8 and 10–12 months while distinguishing native lexical tone contrasts.

Among tonal contrasts, the Bonferroni *post hoc* test (*p* < 0.001) showed that the Tone 1 vs. 3 contrast (*M* = 71.37%, *SD* = 12.63) was easier for infants to discriminate than the other two contrasts, i.e., the Tone 2 vs. 4 contrast (*M* = 61.13%, *SD* = 7.87) and the Tone 2 vs. 3 contrast (*M* = 61.76%, *SD* = 9.76). To further examine the interaction effect between Age and Tone contrast, separate one-way ANOVAs on age effect were run for each contrast. For Tone 1 vs. 3 contrast, 10–12-month-olds (*M* = 82.51%, *SD* = 5.83) performed significantly better than their 6–8-month-old counterparts (*M* = 60.23%, *SD* = 5.69), *F*(1,38) = 149.78, *p* < 0.001, $\eta_{p}^{2}$ = 0.798. Performance of both infant groups in discriminating the Tone 1 vs. 3 contrast was significantly above chance level (percentage of correct response = 50%) at *p* < 0.001, one-sample *t*-test, 6–8 month-old infants, *t*(19) = 8.05; 10–12 month-old infants, *t*(19) = 24.94. However, the perceptual improvement shown for the Tone 1 vs. 3 contrast was not observed in discrimination of the other tone contrasts. For the Tone 2 vs. 4 contrast, older infants (*M* = 62.32%, *SD* = 9.03) did not perform significantly more accurately than younger infants (*M* = 59.93%, *SD* = 6.53), *F*(1,38) = 0.915, *p* = 0.345. Performance of both infant groups was significantly above chance level at *p* < 0.001, one-sample *t*-test, 6–8 month-old infants, *t*(19) = 6.80; 10–12 month-old infants, *t*(19) = 6.10. Further, for the Tone 2 vs. 3 contrast, the performance difference between the older (*M* = 64.75%, *SD* = 12.26) and younger infants (*M* = 58.77%, *SD* = 5.11) was not significant, *F*(1,38) = 4.06, *p* = 0.051, $\eta_{p}^{2}$ = 0.097. Performance of both infant groups was significantly above chance level at *p* < 0.001, one-sample *t*-test, 6–8 month-old infants, *t*(19) = 7.67; 10–12 month-old infants, *t*(19) = 5.38. The result that Tone 1 vs. 3 contrast, the acoustically more distinct contrast, is easier than other tonal contrasts for infants to distinguish, suggests that acoustical salience between tonal contrasts affects the developmental trends of native lexical tone perception.

Results of this experiment showed that, between 6 and 12 months of age, the developmental rates of distinguishing lexical tones varied by tone contrasts. Significant improvement was observed in the Tone 1 vs. 3 contrast; this trend is consistent with previous findings that have shown an increasing sensitivity to native consonants (Kuhl et al., 2006; Tsao et al., 2006; Narayan et al., 2010). However, this developmental trend was less obvious in the other two contrasts, Tone 2 vs. 4 and Tone 2 vs. 3. The results of this experiment reveal a trend that Mandarin-learning infants improve their perceptual sensitivity to discriminate native lexical tones around their first birthdays, but the acoustical salience of tonal contrast would impact the learning rate in developing lexical tones.

## Experiment 2: Perceptual Development of Pitch Contours Among Mandarin-Learning Infants

Results of Experiment 1 revealed that exposure to a lexical-tone language interacts with acoustical salience of lexical tones on the development of lexical tones perception. Pitch contour and height are acoustical cues of lexical tones, but tonal-language speaking adults perceptually weigh pitch contour more than pitch height (Gandour and Harshman, 1978; Gandour, 1984; Chandrasekaran et al., 2010). Would the perceptual improvement of differentiating Tone 1 vs. 3 contrast in Experiment 1 be the result of increased tuning to the familiar pitch contours of this tone contrast among Mandarin-learning 10–12 month-old infants? Experiment 2 explored tonal perception development among Mandarin-learning infants by examining whether 10–12 month-old infants would outperform 6–8 month-old infants in discriminating tonal contrasts with familiar pitch contours. Two sets of tonal contrasts were used in Experiment 2; the pitch height of each lexical tone was the same, and pitch contour difference was the only valid cue to perceptually distinguish the lexical tones. To generate a familiar tonal contrast, one tonal contrast included pitch contours similar to Tones 1 and 3 of Mandarin lexical tones, but the novel contrast included the *inverse* pitch contour of Tone 3 and the non-inverse pitch contour of Tone 1.

### Methods

#### Participants

The participants were 90 Mandarin-learning infants in Taiwan who were tested in two lexical-tone conditions: (1) *familiar lexical-tone contrast*, 7-month-olds (*n* = 23, Mean age = 7.53 months, *SD* = 0.69 months, boys *n* = 10) and 11-month-olds (*n* = 23, Mean age = 11.4 months, *SD* = 0.32 months, boys *n* = 15), and (2) *novel lexical-tone contrast*, 7-month-olds (*n* = 21, Mean age = 7.10 months, *SD* = 0.29 months, boys *n* = 12) and 11-month-olds (*n* = 23, Mean age = 11.13 months, *SD* = 0.25 months, boys *n* = 13). Thirteen additional infants failed to complete the testing procedures because of their inability to pass the conditioning phase. Results of a χ^2^ test on the rate of infants who could not pass the conditioning indicated neither the age nor tone contrast effect reached significance, at 7 months, χ^2^(1) = 0.331, *p* = 0.565, at 11 months, χ^2^(1) = 0.754, *p* = 0.385. The pre-established criteria for inclusion in the experiment were same as in Experiment 1. Parents were paid NT$ 600 for their child participating in the experiment. This study was carried out in accordance with the recommendations of ‘American Psychological Association ethical standards’ and ‘Research Ethics Committees of National Taiwan University’ with written informed consent from all participants. All parents gave written informed consent in accordance with the Declaration of Helsinki.

#### Stimuli, Equipment, and Phonetic Testing Procedure

**FIGURE 3:**
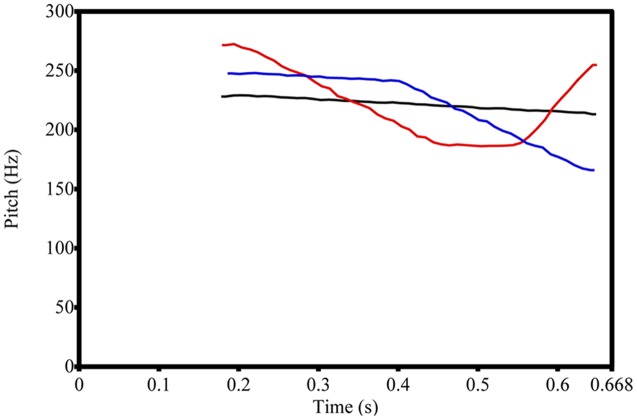
**Pitch contours of speech stimuli in Experiment 2.** Black line = Tone 1 of Mandarin, Red line = tone with familiar pitch contour, i.e., Tone 3 of Mandarin, Blue line = tone with novel pitch contour.

The speech stimuli were Mandarin consonant-vowel syllable ([tɕ^h^i], duration = 668 ms) with three patterns of pitch contour (two familiar tones and one novel tone). These lexical tones consisted of two sets of tonal contrasts in the experiment. For the *familiar* contrast, the pitch contours of speech stimuli were similar to Tones 1 and 3 of Mandarin. To generate the *novel* tone contrast, the pitch contour of one stimulus was similar to Tone 1, but the pitch contour of another stimulus was the inverse of Tone 3, and this pattern did not exist in any lexical tones of Mandarin. **Figure 3** depicts the pitch contours of the speech stimuli. The pitch direction of inverse Tone 3 is generally similar to Tone 4 (falling tone) of Mandarin, but with the later onset of pitch falling. Therefore, combining inverse Tone 3 and non-inverse Tone 1 would generate a novel tone contrast for Mandarin-learning infants. To control the effects of acoustical salience on phonetic discrimination, the average pitch height (mean F0 = 217 Hz) and the vowel formant structures were the same for all speech stimuli, and the *pitch contour* was the only acoustical parameter by which to distinguish lexical tones. To generate more natural stimuli, the speech stimuli were modified from a naturally produced token using the sound-modification software, Praat (Boersma and Weenink, 2011). The testing procedure for the phonetic discrimination was the same as in Experiment 1. For both familiar and novel contrasts, Tone 1 was the background sound in each contrast, but Tone 3 was the target sound in familiar contrast and inverse-Tone 3 was the target sound in the novel contrast.

### Results and Discussion

**FIGURE 4:**
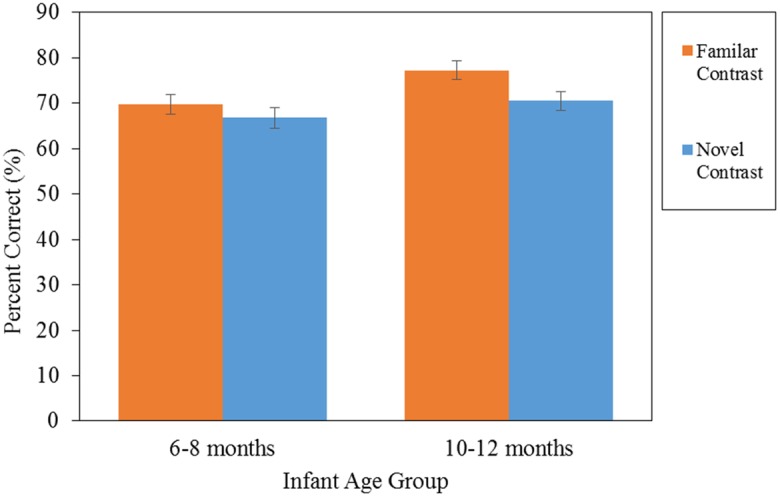
**Results of Experiment 2 on 7- and 11-month-old Mandarin-learning infants distinguishing tonal contrasts with familiar or novel patterns of pitch contours in lexical tones (SE in parenthesis)**.

**Figure 4** displays the percentages of correct lexical tone discrimination at 7 and 11 months of age. The results of a two-way ANOVA (between-subject factor, Age: 7 vs. 11 months; Tonal contrast: familiar vs. novel) showed that older infants (*M* = 73.86%, *SD* = 10.12) performed better than younger infants (*M* = 68.30%, *SD* = 10.72), *F*(1,86) = 6.85, *p* = 0.010, $\eta_{p}^{2}$ = 0.074, and the familiar contrast (*M* = 73.47%, *SD* = 11.95) was easier than the novel contrast (*M* = 68.71%, *SD* = 8.78), *F*(1,86) = 5.08, *p* = 0.027, $\eta_{p}^{2}$ = 0.056. The Age × Contrast interaction effect is insignificant, *F*(1,86) = 0.801, *p* = 0.373. However, given the *priori* hypotheses for a lack of tone contour effect at 7 months, and the contour preference emerging at 11 months, planned comparisons (simple effects tests) were conducted to verify the prediction that tone discrimination varies by pitch contour within each age group. At 7 months of age, infants performed similarly in discriminating both familiar (*M* = 69.70%, *SD* = 11.93) and novel (*M* = 66.78%, *SD* = 9.26) tone contrasts, as indicated by a planned comparison, *t*(42) = 0.901, *p* = 0.373, *d* = 0.274. Both 7-month-old infant groups performed above chance level at *p* < 0.001, one-sample *t*-test, familiar contour group, *t*(22) = 7.92; novel contour group, *t*(20) = 8.30. In contrast, at 11 months of age, infants were more accurate in distinguishing the familiar tone contrast (*M* = 77.25%, *SD* = 10.95) compared to the novel tone contrast (*M* = 70.48%, *SD* = 8.12), *t*(44) = 2.38, *p* = 0.022, *d* = 0.702. The performance of both 11-month-old infant groups was above chance level at *p* < 0.001, one-sample *t*-test, familiar contour group, *t*(22) = 11.94 and novel contour group, *t*(22) = 12.10.

The results of Experiment 2 revealed that the improved accuracy in distinguishing lexical tones between 6 and 12 months of age is evident with a familiar tone contrast that contains similar pitch contours to native lexical tones, but not with a novel tone contrast whose patterns of pitch contour does not exist in the native lexical tones. Since pitch contour was the only acoustical cue for infants to distinguish lexical tones, and the performance advantage of familiar tone contrast was observed only among older infants, the results suggest that Mandarin-learning infants perceptually fine tune to the pitch contours of the lexical tones in their native language around 10–12 months of age.

## Experiment 3: Development of Non-Native Lexical Tone Perception

Results of Experiments 1 and 2 revealed that Mandarin-learning infants develop better sensitivity in discriminating the Tone 1 vs. 3 contrast around 12 months of age. However, to fully address the issue that listening to a tonal language shapes language-specific perceptions of lexical tones in early infancy, it is essential to examine whether the infants learning a non-tonal language also change their sensitivity for perceiving lexical tones. Perceptual decline in distinguishing lexical tones of a foreign language was repeatedly reported among non-tonal language learners after 9 months of age (Mattock and Burnham, 2006; Liu and Kager, 2014; Cabrera et al., 2015).

In the present experiment, English-learning infants were tested with the Tone 1 vs. 3 contrast for which a developmental trend was clearly shown among Mandarin-learning infants in previous experiments. Therefore, the results of this experiment would be compared with those of Mandarin-learning infants in Experiment 1, and this experiment recruited only 6–8 and 10–12 month-old American infants.

### Method

#### Participants

This experiment included 6–8-month-old (*n* = 19, mean age = 7.40 months, *SD* = 0.23 months, boys *n* = 9) and 10–12-month-old (*n* = 21, mean age = 10.87 months, *SD* = 0.17 months, boys *n* = 9) English-learning infants. Seven additional infants failed to pass the conditioning and were excluded from the final data analysis. Results of χ^2^ test on the rates of infants who could not meet the criterion of conditioning phase in the tone discrimination procedure indicated neither the age nor language effect reached significance, at 6–8 months, χ^2^(1) = 0.168, *p* = 0.681, and at 10–12 months, χ^2^(1) = 0.138*, p* = 0.711. The pre-established criteria for inclusion in the study were the same as those employed in the previous experiments. Parents were paid US$ 10 for participating in this experiment. American infants were recruited through the database of names of the Infant Studies Subject Pool (ISSP) at the University of Washington. This study was carried out in accordance with the recommendations of ‘American Psychological Association ethical standards’ and ‘IRB of University of Washington’ with written informed consent from all participants. All parents gave written informed consent in accordance with the Declaration of Helsinki.

#### Stimuli, Equipment, and Phonetic Testing Procedure

As in Experiment 1, the lexical tone stimuli were naturally produced Mandarin tokens of Tone 1 and Tone 3. The testing procedure for the phonetic discrimination was the same as in Experiment 1.

### Results and Discussion

**FIGURE 5:**
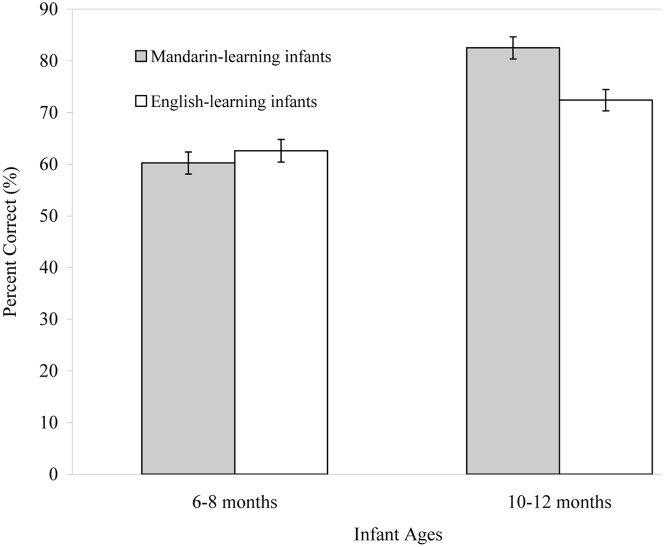
**Mean percentage of the correct responses (+ *SE*) of English- and Mandarin-learning infants in the discrimination of a Mandarin lexical tone contrast (Tone 1 vs. 3) at 6–8 and 10–12 months of age.** Mandarin-learning infants were tested in Experiment 1 and English-learning infants were tested in Experiment 3.

The results of the English- and Mandarin-learning infants on the discrimination of the Mandarin Tone 1 vs. 3 contrast are illustrated in **Figure 5**. As with the data collected from the Mandarin-learning infants in Experiment 1, the percentage of the correct responses of English-learning infants was examined using a 2 (Language background) × 2 (Infant age) ANOVA to examine the development of tone perception. Results showed that the older infants from both language backgrounds were generally more accurate than their younger peers in discriminating tone contrast, *F*(1,76) = 56.65, *p* < 0.001, $\eta_{p}^{2}$ = 0.427. The language background factor was not significant, *F*(1,76) = 3.32, *p* = 0.072. Performance of English-learning infants at both ages was above chance level at *p* < 0.001, one-sample *t*-test, 6–8 month-old group, *t*(18) = 3.82; 10–12 month-old group, *t*(20) = 10.48. However, a significant Age × Language background interaction, *F*(1,76) = 8.60, *p* = 0.004, $\eta_{p}^{2}$ = 0.102, was observed, which indicated that improved accuracy in distinguishing lexical tones varied by the infants’ language backgrounds.

To further examine the developmental trajectories of perceiving lexical tones in infancy, separate one-way ANOVAs were run. The results of Experiment 1 showed that the older Mandarin-learning infants discriminated the Tone 1 vs. 3 contrast more accurately than the younger infants. This perceptual improvement was also observed for the non-native lexical tones discriminated by the older English-learning infants (*M* = 72.38%, *SD* = 9.78), who were more accurate than their younger counterparts (*M* = 62.59%, *SD* = 14.37), *F*(1,38) = 6.45, *p* = 0.015, $\eta_{p}^{2}$ = 0.145. This result led to the following question: “Is language-specific tone perception apparent at either younger age around 6–8 months or at a later age around 10–12 months?” At the age of 6–8 months, English-learning infants performed similarly to Mandarin-learning infants at the same age, *F*(1,37) = 0.47, *p* = 0.499. In contrast, at 10–12 months, Mandarin-learning infants outperformed English-learning infants in detecting lexical tone differences, *F*(1,39) = 16.02, *p* < 0.001, $\eta_{p}^{2}$ = 0.291. Results of this experiment revealed that language-specific lexical tone perception is not apparent among infants aged between 6 and 8 months, but it is apparent around the age of 10–12 months.

Infants’ performance in discriminating non-native lexical tone contrasts was reduced between 6 and 9 months of age (Mattock and Burnham, 2006; Mattock et al., 2008; Yeung et al., 2013; Liu and Kager, 2014). However, the results of the present experiment revealed a different trend: an improved sensitivity in the perception of non-native lexical tones after 10 months of age. The result that English-learning 10–12 month-olds outperform younger English-learning infants in the discrimination of a lexical tone contrast (i.e., the Mandarin Tone 1 vs. 3 contrast) suggest that the listening experience with specific lexical tones would not be the only mechanism by which infants learn lexical tones. Other abilities of speech perception development, such as detecting prosodic patterns of words and phrases in English (Jusczyk et al., 1999; Soderstrom et al., 2003; Seidl, 2007), might also contribute to the development of lexical tones.

## General Discussion

This study explored two issues related to the development of lexical tone perception in three experiments. The first sought to explore the developmental trends in the perception of native and non-native lexical tones between 6 and 12 months of age, while the second questioned whether infants learning a tone language fine tune to the pitch contour of lexical tones while showing the development of tone perception. The results of Experiment 1 on Mandarin-learning infants showed diverse trends in the discrimination of native lexical tones between 6 and 12 months of age. The *improvement* in distinguishing tonal contrasts was observed only for the Tone 1 vs. 3 contrast, but older and younger infants performed similarly when they were tested with the Tone 2 vs. 3 and Tone 2 vs. 4 contrasts. Results of Experiment 1 revealed both *facilitation* and *maintenance* of discriminating native tonal contrasts, and suggested that the relative complexity of pitch contours among tonal contrasts would influence the learning rates of lexical tones. Experiment 2 utilized speech stimuli with familiar and novel pitch contours of Mandarin lexical tones to explore whether Mandarin-learning infants improved their ability to perceive pitch contours between 6 and 12 months of age, and results showed that the fine tuning to pitch contours was apparent with the familiar tone contrast, but not with the novel contrast. Results of Experiment 3 showed that older English-learning infants outperformed their younger counterparts in perceiving the Tone 1 vs. 3 contrast of Mandarin, indicating an *improvement* in the perception of *non-native* lexical tones. Additionally, 10–12-month-old Mandarin-learning infants were more accurate than their English-learning counterparts in distinguishing Mandarin lexical tones, suggesting that the experience of listening to a tonal language facilitates infants’ ability to form detailed representations of lexical tones around 12 months of age.

On the perceptual development of phonetic segments, studies on consonant and vowel perception have reported an improvement in the discrimination of phonetic segments in infants’ native languages between 6 and 12 months of age (Polka and Bohn, 1996; Kuhl et al., 2006; Tsao et al., 2006; Narayan et al., 2010; Pons et al., 2012). The current study extended these findings on the perception of native phonetic segments to lexical tones, the suprasegmental units in phonology. Results of this study reveal a trend of native tone perception: tonal-language learners exhibit a language-general pattern at 4–6 months of age to discriminate tone contrasts of native and foreign languages (Mattock and Burnham, 2006; Yeung et al., 2013), and infants raised in tonal language elevate their accuracy of distinguishing native tones between 6 and 12 months of age. The improved sensitivity to native tones is only shown for the Tone 1 vs. 3 contrast, but rate of development is relatively slow with regards to the Tone 2 vs. 3 and Tone 2 vs. 4 contrasts. Results of the current study are consistent with previous studies. The current study produced multiple indicators that the rates of developing native tone perception vary with tone contrasts and therefore, with acoustical salience. English-learning infants also improved in discrimination of non-native tone contrasts with relatively large acoustical salience. The multiple trends of discriminating native and non-native lexical tones suggest that a hybrid of attunement and perceptual learning theories (Aslin and Pisoni, 1980) would better account for the interaction effects of language experience and acoustical salience on tone perception development. In addition, the results imply that several mechanisms would facilitate infants to acquire lexical tones.

First, the enhanced ability to perceive acoustical parameters of spoken words between 6 and 12 months of age might help infants tune to valid acoustical features for processing lexical tones of words. The speech stimuli in Experiment 1 did not manipulate the critical acoustical parameters of lexical tones, but the acoustical salience of these tone contrasts varied, suggesting an effect of acoustical salience on the learning rate of native lexical tones. Spectral cues to lexical tones, such as average pitch height and pitch contour, are major acoustical cues to lexical tones (Liu et al., 2007; Chandrasekaran et al., 2010). The pitch contour is the only acoustical cue to distinguish tones in Experiment 2, the results of which showed that older Mandarin-learning infants performed better in the discrimination of tone contrasts with familiar pitch contours (similar to Tone 1 vs. 3 contrast in Experiment 1) than for the tone contrast with novel pitch contours, but that the perceptual ability to distinguish familiar vs. novel tone contrasts was not apparent at younger ages. Therefore, the results of Experiment 2 showed an increasing sensitivity to the pitch contour of native lexical tones between 6 and 12 months of age, supporting the acoustical account of lexical tone perception development. The results of Experiment 3 showing that the 10–12 month-old English-learning infants perform better than younger infants of the same language in distinguishing the acoustically salient tone contrast suggest that the acoustical salience account is also applicable to developmental changes seen with non-native tone perception.

Despite that pitch height and contour of lexical tones are major acoustical parameters of lexical tones, results of these experiments imply that older Mandarin-learning infants differentiate tone contrasts with distinct contours (e.g., Tone 1 vs. 3) by attending to pitch contour difference, but they might extra attend to the initial segment of lexical tones (e.g., the first half) when discriminating tone contrasts with similar contours (e.g., Tone 2 vs. 3 and Tone 2 vs. 4). However, older Mandarin-learning infants are not more effective than younger infants when attending to the onset rather than the whole segment of tone contour when discriminating contour tones. F0 frequency of tone onsets differ for contour tones, but the directions of pitch change in the initial part are very similar. The pitch directions of Tones 2, 3, and 4 in Experiment 1 have similar trends in tone onset (shown in **Figure 1**), and pitch directions of novel tone and Tone 1 in Experiment 2 is almost parallel in the tone onset (shown in **Figure 2**). Therefore, older Mandarin-learning infants would not perform better than younger infants in the discrimination of tone contrasts with similar onset contour. The importance of pitch onset in perceiving lexical tones was reported in Cantonese-speaking 5–6 year-old children when they identified the lexical tones with similar pitch contours (Tong et al., 2014). Future studies might manipulate pitch directions of tone onset to assess the role of perceiving pitch onset in developing native lexical tones between 6 and 12 months of age.

The acoustical account of tone perception development has been proposed (Singh and Fu, 2016), and several infant studies on tonal perception provide supporting evidence. In addition to the current study, the effect of acoustical salience on lexical tone contrasts was observed among infants raised in Singapore learning native lexical tones between 6 and 9 months of age (Fu et al., 2015). One-year-old Mandarin-learning infants were more accurate at distinguishing acoustically more distinct tone contrasts than was the case for acoustically more similar contrasts (Tsao, 2008). The difference of improvement in the sensitivity to detecting musical pitch in 4- and 12-month-old Dutch-learning infants was congruent with the improved performance of lexical tone perception; thus, older Dutch-learning infants performed better than younger infants when discriminating the Mandarin tone contrast, suggesting that the improved ability to perceive acoustical features of pitch contour is essential for developing lexical tones (Chen et al., 2017). In addition to fundamental frequency, the perceptual weights of spectral and temporal modulation cues of speech signals also vary between tonal and non-tonal language speakers (Xu and Pfingst, 2003; Cabrera et al., 2014). Non-tonal language adult speakers rely on the amplitude modulation (AM, the relatively slow variation of amplitude over time) information to recognize lexical tones, while Mandarin speakers utilize frequency modulation (FM, the variation of instantaneous frequency) cues to identify and discriminate lexical tones (Xu and Pfingst, 2003; Wang et al., 2011; Cabrera et al., 2014). In line with studies involving adults, French-learning 10-month-old infants preferred AM cues over FM cues in distinguishing lexical tones, but Mandarin-learning infants of the same age utilized FM cues more than AM cues in tone perception (Cabrera et al., 2015). These studies suggest that acoustical features of lexical tones in infants’ native language affect the learning rates of developing lexical tones in infancy.

Second, another mechanism for developing lexical tone perception would be associated with infants’ ability to process linguistic functions of supra-segmental units, such as word stress and sentence intonation (Singh and Fu, 2016). In tonal languages, lexical tones are the essential elements for constructing syllables, and they function like consonants and vowels in distinguishing lexical meanings of syllables. This phonemic function of lexical tones could result in a developmental trajectory of lexical tones in infancy similar to the trends of consonants and vowels, as reduced accuracy in discriminating lexical tones of a foreign language was reported among non-tonal language learners across 6 and 12 months of age (Mattock and Burnham, 2006; Mattock et al., 2008; Yeung et al., 2013; Liu and Kager, 2014; Cabrera et al., 2015). Results of Experiment 3 showed that, for non-native lexical tones, improved sensitivity was observed when English-learning infants distinguished the Mandarin Tone 1 vs. 3 contrast. Improvement in the perception of non-native phonemes that are not included in the phonetic inventory of infants’ native language is rarely documented among infants aged between 6 and 12 months; nonetheless, this trend of improving non-native lexical tone perception is not entirely unexpected. Recent studies have reported that during the second year of life, infants learning non-tonal languages exhibit either better sensitivity than younger peers (Liu and Kager, 2014) or an ability to distinguish the lexical tones of Mandarin at approximately 18 months of age (Hay et al., 2015, Experiment 3; Singh et al., 2014; Zhao and Hay, 2015).

Besides phonemic functions, lexical tones are supra-segmental units of phonetics that are expressed with speech prosody. Prosodic information of stressed syllables facilitates word segmentation for English-learning infants (Jusczyk et al., 1999), and English-learning infants rely more on prosodic information than on phonotactic cues in word segmentation at approximately 9–11 months of age (Mattys et al., 1999; Johnson and Seidl, 2009). Infants learning non-tonal languages detect the prosody of basic emotions very early in life (Mastropieri and Turkewitz, 1999; Singh et al., 2002), and children’s abilities to utilize emotional prosody to recognize speaker’s emotions behind the words continue to develop during early childhood (Quam and Swingley, 2012). The increasing ability to utilize prosodic information in the perception of words and emotions in English-learning infants might facilitate their efforts to distinguish prosodic features in a foreign language; it also reveals a developmental trend of non-native tone perception that is different from the trend of perceptual decline for consonant and vowel contrasts of foreign languages.

The intonation of a sentence is one of the prosodic cues used to differentiate statement and question sentences. Pitch direction in certain lexical tones in Mandarin are similar to those of sentence intonations in English. The rising pitch direction of Tone 2 is similar to the intonation of questions and the falling pitch direction of Tone 4 is similar to the intonation of statements. Dutch-speaking adults were more attentive to pitch movement of Tone 2 and Tone 4 when intonations served the post-lexical function, e.g., differentiating statements and questions (Braun and Johnson, 2011). In future studies, exploring whether English-learning infants exhibit performance changes when distinguishing Tone 2 vs. Tone 4 between 6 and 12 months of age would help to test the assumption that improving prosodic perception facilitates the development of perception of non-native lexical tones.

Would both developmental mechanisms of lexical tones compete with each other or work together for tone perception development in infancy? The present finding that 10–12-month-old Mandarin-learning infants are more accurate in detecting tonal differences of Mandarin than English-learning infants of the same age suggest that improvement in tuning to language-specific lexical tone acoustics would combine with the improving ability to perceive speech prosody for tone-language learning infants in developing their perception of lexical tones.

## Conclusion

Multiple trajectories to the development of distinguishing native lexical tone contrasts were found in Mandarin-learning infants between 6 and 12 months of age, and improving perceptual sensitivity was apparent in the Tone 1 vs. 3 contrast, the contrast with greater acoustical salience. In addition, perceptual advantage of Mandarin-learning infants utilizing familiar pitch contours was found among 8–10 month-old infants. For non-native lexical tones, older English-learning infants outperformed their younger counterparts in the discrimination of Mandarin tone contrast. In addition, 10–12-month-old Mandarin-learning infants distinguished lexical tones more accurately than English-learning infants at the same age. Therefore, this paper suggests that both the fine tuning to acoustical features of lexical tones and improving ability in processing prosodic features of supra-segmental units contribute to the development of lexical tone perception before infants’ first birthdays.

## Author Contributions

F-MT conducted data collection and prepared the manuscript.

## Conflict of Interest Statement

The author declares that the research was conducted in the absence of any commercial or financial relationships that could be construed as a potential conflict of interest.
